# RETRACTED: Fatima et al. Design and Evaluation of Solid Lipid Nanoparticles Loaded Topical Gels: Repurpose of Fluoxetine in Diabetic Wound Healing. *Gels* 2023, *9*, 21

**DOI:** 10.3390/gels12080672

**Published:** 2026-07-27

**Authors:** Farhat Fatima, Mohammad Aleemuddin, Mohammed Muqtader Ahmed, Md. Khalid Anwer, Mohammed F. Aldawsari, Gamal A. Soliman, Wael A. Mahdi, Mohammed Jafar, Abubaker M. Hamad, Sultan Alshehri

**Affiliations:** 1 Department of Pharmaceutics, College of Pharmacy, Prince Sattam Bin Abdulaziz University, Al-Kharj 11942, Saudi Arabia; 2 Department of Community Medicine (SPM), MNR Medical College, MNR Nagar, Fasalwadi Narsapur Road, Sangareddy 502294, Telangana, India; 3 Department of Pharmacology and Toxicology, College of Pharmacy, Prince Sattam Bin Abdulaziz University, Al-Kharj 11942, Saudi Arabia; 4 Department of Pharmacology, College of Veterinary Medicine, Cairo University, Giza 12211, Egypt; 5 Department of Pharmaceutics, College of Pharmacy, King Saud University, Riyadh 11451, Saudi Arabia; 6 Department of Pharmaceutics, College of Clinical Pharmacy, Imam Abdulrahman Bin Faisal University, Dammam 34212, Saudi Arabia; 7 Basic Sciences Department, Preparatory Year Deanship, Prince Sattam Bin Abdulaziz University, Al-Kharj 11942, Saudi Arabia; 8 Department of Pathophysiology, College of Health Sciences, AL-Rayan Colleges, Al-Hijra Road, Madinah Al Munawwarah 41411, Saudi Arabia

The journal retracts the article titled “Design and Evaluation of Solid Lipid Nanoparticles Loaded Topical Gels: Repurpose of Fluoxetine in Diabetic Wound Healing” [1], cited above.

Following publication, concerns were brought to the attention of the Editorial Office regarding the integrity and accuracy of images presented in this publication [1].

In accordance with standard journal procedures, the Editorial Office and Editorial Board conducted an investigation that identified indications of inconsistencies in the error bars presented in Figures 5, 7 and 8, and multiple duplications of sub-images within Figure 10 showing different experimental conditions. Although the authors cooperated with the investigation, the explanations and supporting materials provided were not deemed sufficient by the Editorial Board to resolve the identified concerns. As a consequence, the Editorial Board has lost confidence in the reliability of the reported findings and has decided to retract this publication in accordance with MDPI’s retraction policy. This retraction was approved by the Editor-in-Chief of the *Gels* journal.

The authors have been informed of this retraction and have indicated their disagreement with this decision.
